# Cancer-Causing Genes Can Convert Even the Most Committed Cells

**DOI:** 10.1371/journal.pbio.0030276

**Published:** 2005-08-28

Healthy, normal cells follow the rules: don't crowd the neighbors, stick to your own tissue, and die when it's time. When cells no longer observe these regulations, they become cancerous, dividing uncontrollably, pushing out their healthy counterparts, and eventually invading other tissues. All cells appear to have the capacity to become cancerous, but most don't. Just what turns a healthy cell into an outlaw remains uncertain, but cancer researchers Terence Rabbitts and colleagues at the MRC Laboratory of Molecular Biology in Cambridge, United Kingdom, think the genetic changes regularly observed in cancer cells can provide some clues.

Tumor cells commonly exhibit chromosomal abnormalities. One type of aberration occurs when DNA strands break and segments between two different chromosomes are swapped, a process called chromosomal translocation. Depending on where these chromosomal breaks occur, the newly fused DNA can produce novel genes called fusion genes. Fusion genes are the result of explicit chromosomal changes associated with different cell types and result in distinct types of cancers. In human connective tissue cancers (called sarcomas), genetic exchange between Chromosomes 21 and 22 produces the *EWS-ERG* fusion gene; this translocation is thought to initiate tumor formation in undifferentiated “progenitor” cells called mesenchymal cells.

Progenitor mesenchymal cells are long-lived and self-renewing, and can give rise to many specialized cells, including muscle cells, bone cells, and connective tissue cells. Because the *EWS-ERG* fusion gene in humans was observed only in sarcomas derived from progenitor lineages, researchers thought it initiated a differentiation program that transformed uncommitted non-cancer cells into committed cancer cells. [Fig pbio-0030276-g001]

**Figure pbio-0030276-g001:**
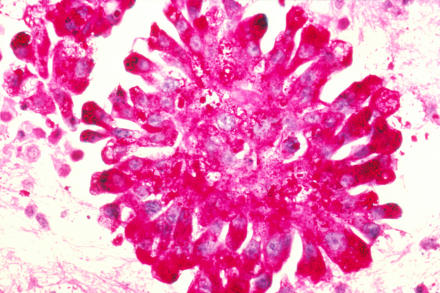
Genetic exchange between chromosomes can cause cells to become cancerous, like these cells from metastasized Ewing's sarcoma (Image: Lance Liotta Laboratory)

To determine whether the Ews-ERG fusion protein could initiate tumorigenesis in other lineages, particularly lineages of committed cells not typical of human cancers, Rabbitts and colleagues genetically engineered mice to express an Ews-ERG fusion protein exclusively in committed B and T immune cells. Mice that expressed the fusion protein developed T cell tumors, demonstrating for the first time that Ews-ERG could cause blood-borne cancers from committed cells, but B cell tumors were not observed. Since the Ews-ERG fusion protein was expressed in both B and T cells in the mutant mice, these results suggest that other factors influence the fusion protein's potential to cause cancer.

These results reveal important information about the way tumors are generated. The cancer-causing effects of the Ews-ERG fusion protein are neither specific to a given tissue type, nor do they exclusively activate tumor cell differentiation in progenitor cells, as originally thought. Rather, *EWS-ERG* may be able to act as a universal cancer-causing gene, inappropriately activating signaling pathways responsible for regulating the cellular lifecycle. These observations, if shown to extend to other known cancer fusion genes, may indicate that the apparent tissue specificity of Ews-ERG and other similar fusion proteins stems from a particular propensity for chromosomal translocations in a given cell type, rather than from the specificity of the resulting fusion proteins.

